# An Assessment of Local People's Support to Private Wildlife Conservation: A Case of Save Valley Conservancy and Fringe Communities, Zimbabwe

**DOI:** 10.1155/2019/2534614

**Published:** 2019-03-03

**Authors:** Given Matseketsa, Billy B. Mukamuri, Never Muboko, Edson Gandiwa

**Affiliations:** ^1^Centre for Applied Social Sciences, University of Zimbabwe, P.O. Box MP167, Mt Pleasant, Harare, Zimbabwe; ^2^School of Wildlife, Ecology and Conservation, Chinhoyi University of Technology, Private Bag 7724, Chinhoyi, Zimbabwe

## Abstract

The long-term survival of a protected area (PA) may depend to a greater extent on the goodwill and support of the people residing around it. This study assessed local people's support for private sector driven wildlife conservation in Zimbabwe, using the Save Valley Conservancy (SVC) as a case. Specifically, the objectives of the assessment were threefold: (i) to establish perceptions on the current nature of the relationship between SVC and people living on its edge, (ii) to ascertain the proximate and underlying causes of local resistance to SVC, and (iii) to identify strategies local people employ to resist SVC conservation efforts. Data were collected through a household questionnaire survey during the month of April, 2018. In addition, photographs showing the nature of vandalism and sabotage imposed on the SVC ecosystem by fringe communities were also collected, as part of evidential data. A multistage sampling method was adopted, and this combined purposive sampling to select study wards: random sampling to select villages and systematic sampling to select households (*n*=71). Our results show that local people rate the current relationship between them and SVC owners as bad, i.e., undesirable interaction. The nature of this perceived bad relationship is attributed to a host of factors, key among them being, lack of wildlife-related benefits and escalation of wildlife-induced costs, which are crucial in determining local community's support for conservation. We conclude that the studied local community's support for private nature conservation is marginal; hence, there is a need for increased efforts by SVC owners to devise realistic incentives including an active engagement of local communities so that they cooperate with conservation efforts.

## 1. Introduction

Protected areas (PAs), whether state or privately owned, have been the mainstay of international conservation strategies since the start of the twentieth century [1]. Until recently, the commonly adopted PA system's preservation model was replicated globally from the American Yellowstone model known as “fortress conservation.” The fortress conservation doctrine was based on the premise that wild species must be preserved by reserving areas and barring people (or at least the wrong sort of people) from living within and using the resources from these areas [2]. In the African context, this fortress conservation indicated a radical departure from the traditional methods of living with nature [3]. More specifically, this ethnocentric conservation strategy viewed native Africans as a clear evil, a “weed” to be removed from the purity of wild nature [4, 5]. However, the question that arises is that if the “fortress” was or is meant to protect natural resources that are within it, what then happens if the protected animals go out? Or does the “fortress” protect those living outside from what is kept within, such as crop-raiding animals, dangerous predators, and diseases endangering livestock and people? [6]. In other words, the phrase “protected area” may be misleading as it means various things to different people. Protected from what and for whom? [7, 8]. Vague, ignorant, incorrect, or evasive replies to such questions can sometimes be highly counterproductive and generate significant conflicts around PAs. Notwithstanding the fact that the gazettement and management of PAs has been a source of disaffection amidst the indigenous communities because of the top-down approach followed in creating and managing them, there is the aspect of the “hidden” costs which were brought forward afterwards such as the loss of economic opportunities, denied access to key livelihood resources and crop and livestock depredation by wild animals [9, 10]. The additional cost of human-wildlife conflict (HWC) has often been seen to stir passionate opposition in already agitated people who feel that these damaging animals are valued more than their lives [11]. This line of reasoning resembles Gillingham and Lee's [12] argument that communities who do not feel a part but at the same time bear the costs of conservation are expectedly unsupportive of conservation. In scenarios where wildlife-induced damages to human property and life are neither controlled nor compensated, opposition toward conservation and wildlife resources become entrenched [13, 14].

Thus, in the African context, conservation particularly enforced through the forced establishment of some PAs has been thought of simply as a protective “locking away” of resources by a powerful elite who have time to enjoy the beauty of nature, while exhibiting a selfishness and anticommunity development agenda [15]. Following the above argument, Carruthers [16] stresses that PAs from the outset were perceived as “white inventions, serving as instruments of dispossession and subjugation” in which Africans were nonpartners who were neither able to continue their traditional subsistence lifestyles in conserved areas, nor were fully co-opted into the system of Western conservation imposed on them. This alienation of African communities, especially those living at the edge of PAs, turned potential conservation allies into adversaries. Consequently, many PAs in Africa to date share a common salient feature: historical poor public relations and minimal support from local communities [17]. Mkomazi Game Reserve (now a national park) in Tanzania is an archetypal case in the field of conservation studies, showing how a PA can face outright opposition [18]. In fact, Norgrove and Hulme [19] made an observation that the relationships between PAs and people are best understood as struggles in which “PA neighbours” use overt and covert “weapons of the weak” to challenge the hegemony of conservation. This is problematic as Holmes [20] insists that, when people are disgruntled, they enthusiastically resist, and consequently, conserving the resources of PAs in the face of opposition is difficult and costly. Further to this, Cavanagh and Benjaminsen [21] clearly stated that local societies are not passive victims but “powerful and potentially transformative agents” who frame their resistance by interpreting their own experiences of marginalization and injustices.

More recently, in particular, in the third world, there has been a gradual realization that PAs cannot survive without support of their neighbours [22]. For example, the opening speech of the president of the World Conservation Union (IUCN) to the Fourth World Parks Congress stated that *“quite simply, if local people do not support PAs, then PAs cannot last”* [23]. Furthermore, Barrow and Fabricius [24] state that “ultimately, conservation and PAs must either contribute to national and local livelihoods, or fail in their biodiversity goals.” Thus, it is increasingly recognized that the fate of PAs is tied to local support [25]. The above recognition has led to a paradigm shift marked by people-centered conservation strategies, policies [26], and most importantly, the valuing of local ecological knowledge in areas where human communities live inside and around PAs [27]. In Zimbabwe, the shift has seen the implementation of the Communal Area's Management Programme for Indigenous Resources (CAMPFIRE) as a mechanism to improve relations between PAs and their neighboring communities [28]. Also, the adoption of the Community-Based Natural Resource Management (CBNRM) as the overall framework guiding or around which conservation of wildlife and other natural resources is organized, inclusive of local communities, has been witnessed in Zimbabwe and Southern Africa as a whole [29]. Key strategies for encouraging local cooperation include devolution of decision-making on resource management and governance [30], benefits, and resource rights to local levels in anticipation for positive reciprocal action. Privately owned PAs or conservancies have adopted mantras or concepts such as “moving beyond the fences” to highlight the desire to both involve and ensure that communities benefit from wildlife [31]. Despite these clear attempts by PAs management to secure local support, resistance appears to continue or even escalate. So far, what perpetuates the resistance particularly in the Save Valley Conservancy (SVC) context remains ambiguous. Thus, an exploration of the factors that arouse a strong desire in people to resist conservation efforts is necessary as a precondition to alleviating resistance wracking PAs. In this present study, the term “resistance” means the violation of conservation institutions driven either by need or by deliberate distrust and hostility to what are commonly viewed as external and illegitimate authorities governing conservation territories [32]. The objectives of this present study were to (i) establish perceptions on the current nature of the relationship between SVC and people living on its edge, (ii) ascertain the proximate and underlying causes of local resistance to SVC, and (iii) identify strategies local people employ to resist SVC conservation efforts.

### 1.1. Theoretical Framework

This study utilized Brockington's [18] principle of local support and Scott's [33] theory of everyday resistance. The former states that, if individual PAs are to have any long-term continuity as institutions and if they are to be effective in preserving the biodiversity contained within them, then local people must support them. Discontented local people will resist PA regulations, protest against them, refuse to cooperate with authorities, and not participate in their plans. This will consequently undermine both the institution of a PA and the health of the biodiversity contained within it. The principle has an interesting position in conservation strategy, discourse, and practice as it is analogous to the concept of conservation justice which dictates that local communities are entitled to receive fair treatment and meaningful involvement in conservation endeavours [34]. The latter is premised on the underlying assumption that poor grass roots actors resort to everyday forms of resistance also termed “weapons of the weak” when open confrontation with powerful actors carries the real prospect of a massive retaliatory response. Hence, for this present study, this theory is of utility as it offers better lenses to uncover and understand local acts of resistance, as the current displays of vandalism and sabotage posing significant impacts on SVC's faunal resources are mostly covert and anonymous in nature (i.e., elusive snaring of terrestrial mammal species, cutting and stealing of perimeter fence, unauthorised bushfires, feigned compliance, and noncooperation via illegal grazing). Hence, the theory in question offers a fresh perspective of productively assessing PA-community relations, as it makes conservation practitioners more aware of the forms local resistance can take and the diverse meanings and intentions embodied. Further, the theory reveals that resistance is a tactic utilized by the weak to contest oppression. This implies that local villagers resisting SVC could simply be a call for a more socially just conservation. Thus, if conservation practitioners endorse their call, conservation is eventually made better for both biodiversity and those who live close to PAs.

## 2. Materials and Methods

### 2.1. Study Area

**Figure 1:**
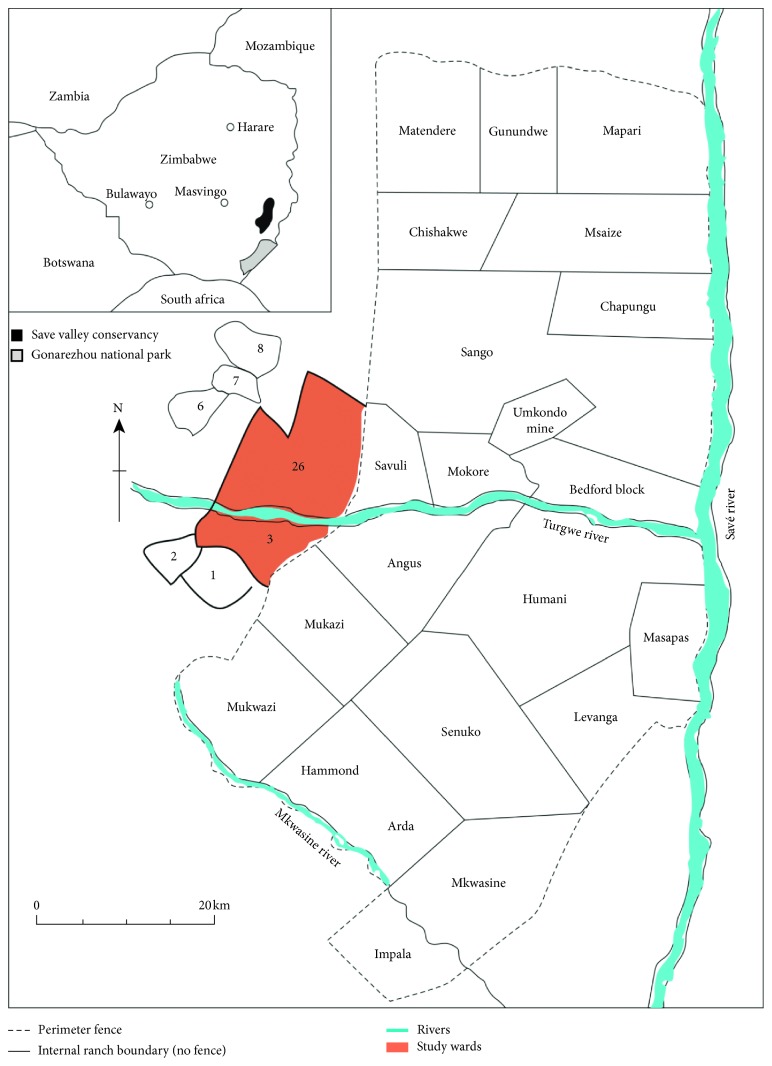
Location of the study communities, i.e., Wards 3 and 26 adjacent to the southwestern SVC, Zimbabwe.

The study was conducted in two local communities falling under the Bikita district, adjacent to the southwestern border of SVC, southeastern Zimbabwe (Figure 1). Communal areas in Zimbabwe are divided into administrative units of villages. Six or seven make a ward or community [35]; hence, in this study, we focused on two communities: Ward 3 and Ward 26. SVC spans an area of 3400 km^2^ (however, during 2000 and 2001, SVC was affected by the onset of the land reform programme such that some of its properties, i.e., Angus, Masapas, Levanga, and Senuko occupying the Southern half, were invaded by subsistence farmers). Up until April 2014, it was a cooperatively managed private wildlife area, but in the month of May 2014, it was placed under the custodianship of the Zimbabwe Parks and Wildlife Management Authority (ZPWMA). The conservancy is located in agroecological region V which is a semiarid area in the southeast Lowveld of Zimbabwe. Its southern boundary is approximately 45 km northeast of Chiredzi town while the Save River and Sangwe communal lands mark its eastern boundary. Its northern boundary lies not far from Birchenough Bridge and its western boundary being formed by a resettlement scheme on land of the former Devuli Ranch and to the South by Matsai Communal area. It is located in Masvingo Province and covers two districts which are Chiredzi and Bikita. It is surrounded by three other districts which are Zaka, Buhera, and Chipinge. SVC is bordered primarily by high-density communal land (of between 11 and 82 people per km^2^) [36], with some commercial agriculture to the south and east. At national level, the average national density for communal areas is 33 people per km^2^ [37].

### 2.2. Data Collection

A multistage sampling technique was adopted due to the nature of the sampling frame, to select the sampling units. The first stage involved purposive sampling following Patton [38]. As a result, two wards (3 and 26) were selected from a total of seven wards. Purposive sampling was found suitable as study communities were in close proximity to SVC boundary, and therefore believed to have much interaction with the PA. The second stage involved simple random sampling for village selection, and this resulted in five (5) out of 11 villages being selected out of the two wards. The villages selected were Matsai, Villages 24, 26, 27, and 31. The third stage involved systematic sampling which resulted in the selection of 71 households (representing 20% of all the village households); this was ensured by picking and interviewing every second household from village registers. Out of the 71 respondents, 79% were males (*n*=56) and 21% were females (*n*=15). The age of the respondents ranged from 18 to >60. About 18% (*n*=13) of the respondents were between 18 and 25 years, 39% (*n*=28) were between 26 and 39 years, 25% (*n*=18) were between 40 and 59 years, whereas 17% (*n*=12) were more than 60 years.

Data collection was conducted in April 2018, using an interview-administered questionnaire. The questionnaire included both open-ended and closed-format questions. Fixed response questions were used to ensure precision of responses, whilst open-ended questions were also included to tap into the views of the villagers and glean more information on the subject of interest. For example, the questionnaire addressed some of the following questions: (i) can you describe the nature of your relationship with the SVC owners and the main factors mediating the relationship? (ii) Do you have any grievances or are you unhappy about something with the conservancy? Incorporating open-ended questions in a questionnaire in ecology has also been advocated for by White et al. [39] who argues that well-designed open questions may provide data of equivalent precision to closed-format ones. All interviews were conducted by the first author with the aid of a field assistant who was selected from the local community and made initial contact in each village with the local village leaders to seek permission. The interview sessions lasted between 15 and 25 minutes. Further, field observations including photography were used as a complementary method to have a visual appreciation of the nature of vandalism and sabotage upon the SVC ecosystem by fringe communities. The method provided an insight into the realities on the ground and also helped in the verification and interpretation of data collected through the social survey. The method of photography has been used in socioecological research elsewhere in the Gonarezhou ecosystem, southeastern Zimbabwe [40].

### 2.3. Data Analysis

Descriptive statistics were used to summarize quantitative data sets from house-hold questionnaires. A nonparametric test, i.e., Kruskal–Wallis chi-squared (*χ*^2^), was also used to determine whether given responses on the nature of relationship locals have with the SVC, proximate and underlying causes of resistance, strategies employed to resist SVC conservation efforts differ across the villages using the Statistical Package for Social Sciences (SPSS) version 20 for Windows (IBM SPSS Inc., Chicago, USA). A *p* value < 0.05 was deemed significant.

## 3. Results

### 3.1. Perceptions on the Prevailing Nature of SVC's Relationship with Neighboring Communities

**Figure 2:**
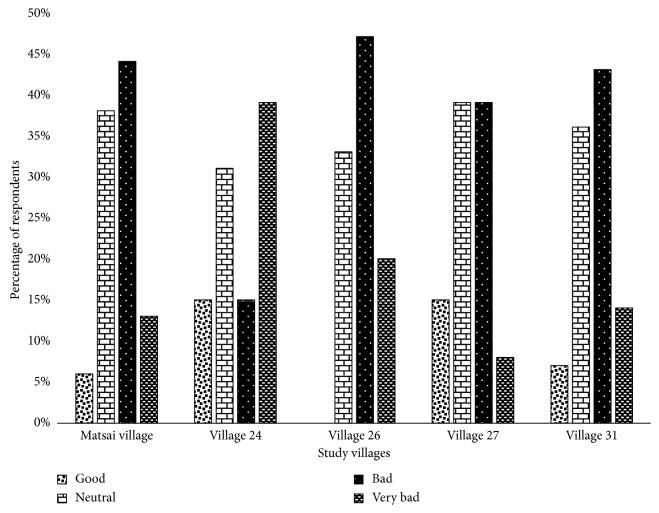
Communities' views of their relationship with the SVC. Note: good means that the interaction between SVC authorities and community members is desirable; neutral means that communities perceived their relationship with SVC authorities to be impartial; bad means interaction undesirable. Villages 24, 26, 27, and 31 fall under Ward 26, and Matsai village falls under Ward 3.

PA-community relationships are dynamic and largely influenced by changing circumstances. Respondents reported mixed perceptions on their relationship with the SVC. In Village 26, the majority of the respondents (*n*=7; 47%) claimed their relationship with the SVC to be bad, whilst a minor proportion (*n*=1; 6%) of the respondents in Matsai village rated the existing relationship as good (Figure 2). There was no significant difference (KW*χ*^2^ = 2.122; df = 4; *p* > 0.05) on the nature of relationship locals have with the SVC across the villages.

The mixed perceptions on the relationship between neighboring communities and the SVC was further evidenced by the majority of respondents, 69% (*n*=49) who indicated that they were anti-conservation (study participants claimed that there was an increase in the number of problems caused by SVC's existence to adjacent communities such as crop and livestock depredation), while 31% (*n*=22) of the respondents claimed to actively support wildlife conservation in the SVC. There was no significant difference (KW*χ*^2^ = 5.217; df = 4; *p* > 0.05) in the views of local communities with regard to claims to support the SVC across villages.

### 3.2. Proximate and Underlying Drivers of Local Resistance to Biodiversity Conservation in SVC

Respondents have different reasons for resisting biodiversity conservation in the SVC. Factors that were reported to spark resistance based on respondents' views were classified into two categories, namely, proximate (the more immediate factors) and underlying (deep-seated factors). Identified factors included the lack of wildlife-related benefits (*n*=11; 22%), poor control of damage-causing wild animals (*n*=7; 14%), escalation of wildlife-induced costs or conflicts (primarily referring to the damage caused by wild animals to crops and livestock) (*n*=13; 27%), distrust for SVC owners and workers (*n*=5; 10%), limited and irregular communication between SVC-management and adjacent communities (*n*=6; 12%), lack of compensation for losses from wildlife (*n*=5; 10%), and the lack of community participation in wildlife conservation (*n*=2; 4%) (Table 1). Overall, there was no significant difference (KW*χ*^2^ = 2.005; df = 4; *p* > 0.05) in the reasons behind resistance respondents displayed across the villages.

### 3.3. Tactics Adopted by Respondents to Resist SVC

**Figure 3:**
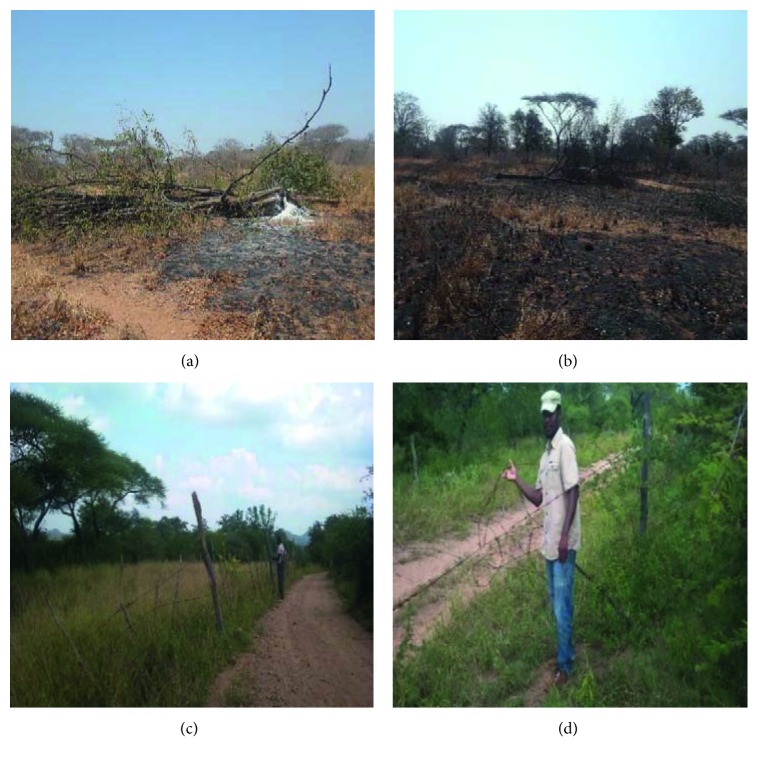
(a, b) Unauthorised fire-setting with a malicious intent in one property which is part and parcel of the SVC during the study period; (c, d) field assistant alongside the first author physically verifying the vandalism of perimeter fence by fringe communities. Photo credits: L. Phikelele (fire-setting) and G. Matseketsa (perimeter fence), 2018.

Study respondents in communities living adjacent to the SVC use several methods to attenuate their hardships and express their discontent. About six strategies are used in confronting nature conservation in the SVC ecosystem, namely, poaching of terrestrial mammal species (*n*=19; 39%), cutting and stealing of perimeter fence (*n*=8; 16%) (Figure 3), feigned compliance (*n*=7; 14%), collaborating with external poachers (*n*=3; 6%), non-cooperation via grazing trespass (*n*=6; 12%), and not able to actively oppose the SVC (usually because of old age or fear of SVC authorities and punishments) (*n*=6; 12%) (Table 2). There were no significant differences (KW*χ*^2^ = 3.929; df = 4; *p* > 0.05) on the strategies used to resist SVC across the villages.

## 4. Discussion

This study provided an opportunity for the first time to examine factors besetting local residents' support for nature conservation. The SVC provides an excellent case for exploring these dynamics of conflict and social resistance as it has endured sustained opposition from local human communities in designating and managing it. McCleave et al. [41] found that several factors are often at play in shaping the relationship between a park neighbor and a park in New Zealand. Thus, a more nuanced understanding of the factors that impede positive PA-local population relationships is critical for fostering sustainable conservation relationships. Our results show that the current relationship between SVC and local inhabitants is dysfunctional, and it is bad (Figure 2). Local people's relationship to the SVC is complex; however, on a preliminary basis, the existence of an uneasy and bitter relationship can be attributed to the several factors uncovered by the present study. From respondents' perspective, a host of factors were raised as important in shaping how they relate with the PA; these determinants are discussed in subsequent sections.

### 4.1. Determinants of Community Resistance

The present study demonstrated that respondents residing near SVC hold different reasons for resisting its conservation efforts. Results show that there were seven main factors perceived by local people to animate and strengthen resistance toward wildlife conservation in the SVC. Stated factors include limited benefits from wildlife, poor control of damage causing wild animals (no/delayed response to HWC incidents), escalation of wildlife-induced costs/conflicts, local distrust for PA officials, insufficient communication between SVC-management and local human communities, lack of compensation for loss accrual, and the lack of community participation in wildlife conservation (Table 1). The factors conveyed by respondents tend to be similar across the villages at first glance. However, a critical look at the results reveals that the lack of benefits and escalation of wildlife-induced costs are key drivers for the passionate opposition toward wildlife conservation in the SVC across villages. These findings are in synchrony with previous studies on the topic. In Laikipia district, Kenya, people were willing to express devotion for wildlife and wildlife areas if they did not suffer losses to wildlife continuously and later on derive minimal benefits from it [42]. It is believed that, if a balance is struck between cost and benefit, accrual opposition for nature conservation is reduced. According to Gillingham and Lee [12], local people who disproportionately bear the cost of protection and feel “excluded” cannot be expected to provide the needed support if the costs of doing so outweigh the benefits they derive. In short, local communities do whatever maximizes their own profit and that positive reciprocity is contingent upon receiving benefits [43]. The absence of discernable benefits and escalating costs justify local communities' continued resistance toward the SVC and nature conservation at large. On top of this, in a broader sense, the escalation of conservation-related costs, i.e., crop raiding, can often make the difference between hunger or food sufficiency. In other words, costs incurred can breed or exacerbate poverty in local communities, further creating an acrimonious conservation climate as in this case.

Moreover, lack of control of damage causing wild animals was also reported by respondents as a strong reason for the opposition displayed. This confirms the observation by Ayivor et al. [11] that anything that threatens a source of livelihood in local people inevitably erodes support for conservation and garners resistance. The issue of lack of control (referring to the authorities failure to control problematic wild animals/land owners less responsive) of damage-causing wild animals is quite rampant in the SVC context. The SVC subsists on hunting tourism to self-finance its operations, and so the killing of wild animals on the basis of problem animal control is regarded as a bad business ethic in the sphere of safari hunting. Similarly, this phenomenon has also been observed by Dzingirai [44] in the CAMPFIRE context. However, as in this case, when local communities feel that authorities elevate biodiversity conservation over human welfare issues, they take matters into their own hands, eliminating unwelcome animals [45]. In other words, when local communities feel that both governments and conservation stakeholder's value wildlife more than their lives, livelihoods or their aspirations, retaliation, and opposition to conservation initiatives can be swift and uncompromising [13, 14].

Distrust or lack of trust between the SVC and neighboring communities is a key determinant of resistance to conservation efforts. A significant proportion of respondents highlighted distrust for SVC authority. Trust has been identified as an important element of multiple forms of natural resource management processes and outcomes [46]. For example, in a study of national parks in the United States and Ecuador, trust in PA authorities proved to be a key predictor of compliance with park regulations, with distrust predicting noncompliance [47]. Trust held by community members for natural resource agencies has also been shown to increase public approval of management decisions and minimize resistance to planning efforts [48]. Conversely, local communities lack trust in SVC authorities, hence the dissention. This lack of trust can be primarily linked to SVC members' ability to over promise and under deliver. Upon the creation of the SVC, a fence was constructed fencing out local peasants from key livelihood resources, and white shareholders of SVC convinced the peasants that the erection of a fence was a legal requirement for the successful running of hunting tourism operations and that the benefits will surely flow over the fence into the communities [49]. Surprisingly, to this date, benefits are yet to materialize. If promises take as long to materialize, they lose their persuasive appeal, especially if there are no clear indications that things will change for the better soon. Moreover, lack of compensation for losses incurred was also identified as one other factor that provokes a strong sense of hatred, resentment, and opposition in fringe community members. The result is consistent with findings by Mariki [50] who in the Kilimanjaro National Park, Tanzania, discovered the accrual of conservation costs and lack of compensation, sparks hatred, resentment, and ultimately resistance toward conservation. Hence, it has been seen that the provision of compensation for wildlife damage and fair benefit sharing can strengthen local people support for wildlife conservation [50].

Study findings show that limited and irregular communication between PA authority and locals and the lack of community participation in wildlife conservation are key factors, engendering resistance in SVC-adjacent dwellers. These results correspond with findings of previous studies. For instance, a remarkable study by Mutanga et al. [51] focusing on four PAs in Zimbabwe, namely, Gonarezhou National Park, Umfurudzi Park, Matusadona National Park, and Cawston Ranch and their surrounding communities revealed that poor communication was an important factor in inspiring negative park-people relationships. Further to that, the lack of community participation in nature conservation degrades good PA-people relations as in this case. Andrade and Rhodes [14] found that local community participation in the PA decision-making process and nature conservation is significantly related to the level of compliance with PA polices.

### 4.2. Strategies That Neighbours to the SVC Employ to Resist Conservation Efforts

In the SVC context, local inhabitants employ several tactics in resisting nature conservation. Stated strategies include poaching of valuable terrestrial mammal species, cutting and stealing of perimeter fence (Figure 3), noncooperation via illegal grazing, feigned compliance, collaborating with external poachers, and not able to actively oppose the SVC/under resourced to do anything (Table 2). Strategies employed in resisting SVC tend to be similar across the sampled villages. However, a thorough examination of findings shows poaching to be the primary and convenient method utilized in expressing dissatisfaction for nature conservation in the SVC across all five study sites, followed by the cutting and theft of game fence and feigned compliance (e.g., agreeing to discard ecologically detrimental behaviors during community conservation meetings but not comply with it), respectively. Poaching in the SVC has been employed in conjunction with the vandalism of perimeter fence; locals cut the fence which they use to make snares, whilst creating pathways through the fence for wild animals to exit out of the PA into the villages where they can easily “poach” them. Holmes [52] and many others suggest the continuation of outlawed livelihood practices by local communities as an assertion of resistance. Poaching practiced in the SVC is quite peculiar in the sense that locals can catch a wild animal on a snare but somehow choose to leave it there to rot. This phenomenon has also been observed where animals are illegally killed in a PA, but no meat, hides, horns, or other benefits taken and the bodies left to rot (for Kenya; [53, 54]; for USA, [55]). It is believed that, when local communities do that, they will be implicitly making a statement that they have a right to kill animals. Moreover, results on poaching still show that some local community members gang up or accomodate poachers coming from outside their communities [54] in Kenya. This collaboration with external poachers can partly be attributed to the existence of a confrontational park-people relationship as in this case. If a good park-people relationship prevails, local inhabitants themselves become allied with PA management in protecting the area from threatening activities or developments.

Moreover, study findings show that illegal cattle grazing is a technique local people employ in expressing their discontent for the conservancy. This research result is in harmony with findings by Neumann [56] on the same topic that reported, in Arusha National Park, Tanzania, as much as acts such as grazing trespass and park encroachment may be attributed to “ignorance” by conservationists and PA authorities; however, in reality, they have multiple meanings and intentions. Thus, they represent more subtle forms that community objections to conservation may take. Accordingly, there is a Maasai proverb which states that “God gave us cattle and grass; we do not separate the things God gave us” [57]. Interestingly, some respondents from the survey indicated that they were not able to actively oppose the SVC/just under resourced to do anything or fight back. This is justified considering that park-people relationships are asymmetrical in nature, where PA authorities are the wielders of power, and local people, the dissident groups [20]. And according to Chan and Satterfield [58], the capacity to resist unjust practices is a function of power such that those with a greater ability to articulate their grievances are also those most likely to achieve desirable outcomes. Overall, findings obtained show that locals have an inclination toward the covert weapons. These do not publicly challenge the PA but they involve “hidden” activities; hence, it is hard to control the elusive snaring of wild animals, cutting and stealing of game fence, feigned compliance, and bushfires. Holistically, present study findings are in line with results obtained by others. Other studies reveal that people resist conservation by destroying the resources they once valued due to changes in tenure rights [59], fire-setting [60], destroying PA infrastructure [61], and tree cutting [59]. It is concluded that continued hostility from local people is clearly counterproductive to sustainable conservation efforts [62].

## 5. Conclusion and Recommendations

The present study identified and presented the factors that constrain local support for wildlife conservation in the SVC context. Based on our results, it can be concluded that the existing relationship between edge communities and the SVC is bad (not cordial). Local human communities perceive the SVC as a source of much of their anguish. As evident from the results of this study, a multiplicity of factors was brought to light as key in fueling resistance in fringe community members. As a result, local people's support for wildlife conservation is fast eroding, whilst resistance mounts a situation that undermines SVC's long-term ecological character (as it turns into a “paper park,” i.e., only protected on paper). PA neighbours have developed a sophisticated armoury of methods to pursue their livelihood goals and resist the conservation agenda promoted by the SVC and its allies. Thus, the situation around the SVC is a clear-cut hegemonic/counterhegemonic struggle between PA authorities (fighting for the prevailing conservation ideology) and PA neighbours (fighting for or negotiating their right to a modest or decent way of life).

Thus, on the basis of data from this study, local opposition can be neutralized by a number of actions such as (i) the creation of a formal and meaningful relationship (not a “paper partnership” as reported by Corbett [63]) with the neighboring communities, in order to stimulate a more positive form of reciprocity toward wildlife conservation. (ii) Putting in place formalized benefit sharing mechanisms to ensure a steady flow of benefits to local people living on the edge. This is critical as Matseketsa et al. [43] argue that people living in close proximity to PA edges often avoid costs and seek benefits. (iii) There is need to document the economic, social, and opportunity costs of SVC on local communities, thus creating inventories. These inventories can support the development of conservation strategies to minimize the burden of SVC on local villagers while sustainably managing biodiversity. (iv) Under corporate social responsibility and extension work, SVC authorities need to have the capacity to embark on regular outreach programmes to dialogue with community members and to listen to their concerns. Regular dialogue will help to promote mutual trust, reduce acrimony, and curtail conflict situations. (v) PA practitioners could try to move away from labelling all infractions of PA regulation as criminality and irrationality and to recognize and address this vibrant everyday sociopolitics to produce policy that is both better for biodiversity and people in the immediate vicinities.

## Figures and Tables

**Table 1:** Proximate and underlying drivers of local resistance to SVC across the villages.

Ward(s)	Village(s)	Proximate			Underlying			
		Lack of wildlife-related benefits	Poor control of damage-causing wildlife	Escalating wildlife-induced costs/conflicts	Distrust for SVC owners and workers	Limited and irregular communication	No compensation for losses from wildlife	Lack of participation in wildlife conservation
3	Matsai	3	1	2	2	1	2	0
26	24	3	0	3	1	2	1	0
	26	2	2	2	1	1	1	0
	27	1	3	2	0	1	1	1
	31	2	1	4	1	1	0	1
	Total (*n*=49)	11 (22%)	7 (14%)	13 (27%)	5 (10%)	6 (12%)	5 (10%)	2 (4%)

Note: contained in this table are numbers of respondents (not bracketed) who provided a response.

**Table 2:** Strategies respondents employ to resist SVC conservation efforts across the villages.

Ward(s)	Village(s)	Poaching of terrestrial mammal species	Cutting and stealing of perimeter fence	Non-cooperation via illegal grazing	Feigned compliance	Collaborating with external poachers	Not able to actively oppose the SVC
3	Matsai	4	3	1	2	0	1
26	24	3	2	1	2	0	2
	26	4	0	2	1	2	1
	27	5	0	1	0	0	1
	31	3	3	1	2	1	1
	Total (*n*=49)	19 (39%)	8 (16%)	6 (12%)	7 (14%)	3 (6%)	6 (12%)

Note: contained in this table are numbers of respondents (not bracketed) who provided a response.

## Data Availability

The data used to support the findings of this study are available from the corresponding author upon request.
